# In vivo enhancement of the MAGE-specific cellular immune response by a recombinant MAGE1-MAGE3-TBHSP70 tumor vaccine

**DOI:** 10.1186/s12935-016-0317-2

**Published:** 2016-06-17

**Authors:** Wang Junwei, Zhan Xiumin, Ye Jing, Yang Shoujing, Li Zengshan

**Affiliations:** The State Key Laborotary of Cancer Biology, Xijing Hospital of the Fourth Military Medical University, Xi’an, 710032 Shanxi China; The Pathology Department, Fourth Military Medical University, ChangLe West Road 17, Xi’an, 710032 Shanxi China

**Keywords:** Immunotherapy, Melanoma-specific antigens, Cytotoxic T cell response, Vaccine

## Abstract

**Background:**

Since cytotoxic T cell (CTL) response is the major cellular type in attacking tumor cells, most immunotherapy targets to manipulate the CTL response. Immunotherapies targeting melanoma-specific antigens (MAGEs), a group of tumor-specific shared antigen, have shown to be promising. Our previous study has shown that MAGE1/TBHSP70 and MAGE3/TBHSP70 could induce a robust immune response against B-16 melanoma cells in C57BL/6 mice. In this study, we used an animal model to further demonstrate MAGEs as a potential immunotherapy target for tumorigenesis in vivo.

**Methods:**

In the current study, we developed a MAGE1/MAGE3/TBHSP70 recombinant protein vaccine and evaluated its protective efficacy against tumor development by challenge vaccine-immunized mice with MAGE-expressing human tumor cell lines in a Hu-PBL-SCID mouse model. The cellular immune reactions were monitored by ELISPOT and cytotoxicity assays.

**Results:**

Splenocytes isolated from vaccine-immunized mice presented potent cytokine secretion capacity and CTL-specific cytotoxic. Vaccine-immunized mice had a significant tumor regression and prolonged survival compared with controls (both p < 0.05). In vitro, rMAGE1-MAGE3-TBHSP70 showed a potent tumor-antigen-specific immune response in both hepatocellular carcinoma and pulmonary carcinoma cell lines.

**Conclusion:**

This newly-developed recombinant protein vaccine may serve as a new immunotherapy for cancer.

## Background

Anti-tumor peptide/protein vaccines have become a promising therapeutic approach since such vaccines enhance the immune response of body towards the specific anti-tumor antigens, and vaccination itself is a relatively safe approach in clinical practice [1, 2]. Melanoma-associated antigens-A (MAGE-A) family is a group of well-characterized cancer/testis antigens (CTA) which could serve as a potential candidates for anti-tumor vaccines based on the fact that MAGEs are tumor specific, are shared by many different kinds of tumor and can be recognized by autologous cytotoxic T lymphocytes (CTLs) [3–6].

Currently, basic research is under way to study the mechanism of anti-tumor immunity and how MAGE vaccines could play a role in anti-tumor treatment, as well as the efficiency and side effects of this therapeutic approach [7–9]. Scientists have been making efforts to optimize the vaccination strategies, including comparing different linked-adjuvants of the vaccine, engineering the regulating gene and combining multiple CTL epitope to enhance the efficiency of vaccination. Previously, we reported that reconstructed MAGE1-TBHSP70 DNA vaccine and recombinant TBHSP70-MAGE3 protein vaccine were sufficient enough in enhancing anti-MAGE tumor immunity [10, 11]. However, transient transfected animal model was used in that study, which had limited clinical application value. Our further investigation suggested that polyvalent immune response is important to maintain a sustained reaction targeting tumor growth. Data from our study and others indicted that the polyvalent immune response can modulate antigen expression on tumor cells and grant tumor cells with capacity of being resistant to a single antigen approach anti-tumor treatment [12]. Hence, it is interesting and necessary to investigate the possibility and efficiency of multiple antigen targeting anti-tumor immunotherapy. Thus, we engineered, cloned and made a recombinant MAGE1-MAGE3-TBHSP70 protein targeting two member of MAGE family proteins and further studied the therapeutic effects of this recombinant vaccine in anti-tumor response using a PBL-Hu-SCID model in this study.

## Methods

### Vectors

The pET28a(+) expression vector (Invitrogen, CA, USA) containing the T7 *lac* promoter was the basic plasmid used for protein expression. The MAGE-1 (NCBI Reference Sequence NM_004988) fragment in CDS sequence (500–1150 bp, 651 bp) was amplified by PCR from the plasmid pcDNA3-MAGE1 [10]. The amplified product was cloned into pET28a(+) downstream of the T7 *lac* promoter containing *Bam*HI and *Sac*I sites. This plasmid was then named as pET-M1. The *Mycobacterium tuberculosis* HSP70 gene was amplified from the plasmid pcDNA3-HSP70 [10]. The amplified product was further cloned into the *Hind*III and *Xho*I sites of the pET-M1, and the product was named as pET-M1H. The MAGE-3 (NCBI Reference Sequence NM_005362) fragment in CDS sequence (792–1154 bp, 363 bp) was acquired from vector pET-MAGE3 [11] using the similar method mentioned above. The amplified product was cloned into pET28a(+) and pET-M1H with *Sac*I and *Hind*III sites. The product was named as pET-M3 and pET-MMH, separately. pET-M3 was also cloned into the *M. tuberculosis* HSP70 gene and named as pET-M3H. All the productions were verified by DNA sequencing (Sangon Co. Lt., Shanghai China). All primer design, sequence blast and analysis were performed by Vector NTI software (Informax Inc., Maryland, USA).

### Expression, purification, and detection

Expression vectors pET-M1H, pET-M3H, pET-MMH and the empty plasmid vector pET28a(+) were transfected into Escherichia coli Bl21 (DE3) plysS competent cells. The recombinant protein expression was performed according to the manufacture manual. Briefly, the culture pellets were analyzed by SDS-PAGE, and were purified by His GraviTrap™ Flow (Amersham Biosciences, USA) column containing pre-charged Ni Sepharose™ 6 Fast. Purified proteins were detected for antigenicity using western blot. Primary antibodies used in western blot were rabbit polyclonal anti-human MAGE1 (ab21472, Abcam, USA) antibody and rabbit polyclonal anti-human MAGE3 (ab38496, Abcam, USA) antibody whose binding site located in the truncated fragments of the expressed protein. The amino acid sequences were analyzed by mass spectrograph.

### Animal models and cell lines

The homozygous C.B-17 scid/scid male mice were purchased from Slaccas Laboratory Animal Co. Lt. Shanghai China (http://www.slaccas.com) and were housed in a micro-isolated environment. The animal protocol met the criteria of the NIH guide for the care and use of laboratory animals. Hepatocarcinoma cell lines HepG2, human hepatocellular carcinoma cell HHCC and pulmonary carcinomas cell line (A549) were carefully selected according to virtue of approximate growth curve, doubling time and percent of apoptosis. To verify specific immune response, MAGE1 and MAGE3 transcription and expression levels in these cell lines were detected using western blot and nest-real time polymerase chain reaction (net-RT-PCR) according to the standard protocols [13].

### Reconstruction of Hu-PBL SCID

The Hu-PBL SCID mouse model was reconstructed according to a previous method with slight modifications [14, 15]. Briefly, the mice were pretreated 1 day prior to Hu-PBL injection with a single dose of lyophilized anti-ASGM1 antibody (Wako Chemicals, USA). Anti-ASGM1 was a rabbit polyclonal Abs that recognized murine NK cells, through which it depleted NK cell activity. Prior to Hu-PBL engraftment, SCID mice were irradiated with 2 Gy gamma irradiation from a ^60^Co linear accelerator. The peripheral blood mononuclear cells (PBMC) was isolated from healthy donor’s blood using Ficoll-Hypaque centrifugation method and then immediately injected (2 × 10^7^ cells in each mouse) intraperitoneally into irradiated mice. The heparinized serum was obtained from the mice by tail vein bleeding at 2 to 3-week intervals after PBMC injection. The human IgG and mice IgG in serum were quantified using enzyme-linked immunosorbnent assays (ELISAs). Normal mouse serum and donor serum served as controls. The mice were eligible for humanized when the concentration of human IgG reached approximately 3 μg/ml. Mouse with IgG reached 5 μg/ml were considered as unsuccessful humanization.

### Vaccination and detection of humoral immunity and cellular immunity in Hu-PBL-SCID mouse mode

A total of six Hu-PBL SCID mice were finally included in each group. Six naive SCID mice (Un-Humanized Mice, UnHuM) were included as a control group. In addition, the PBL of tuberoculosis patient (TB-PBL) was also pooled and tested in vitro. The mice were immunized twice intra-peritoneally (i.p.) at a 2-week interval with 200 pmol of the purified recombinant proteins diluted in 100 μl PBS (0.1 M, pH 7.3). The PBL of tuberoculosis patient (TB-PBL) was incubated with TBHSP70 an hour in vitro at 24 h interval for the study of non-specific response of vaccine in humanized mouse model.

Heparinized serum was obtained by tail-vein bleeding 1 week after the second vaccination. 96-well plates were coated with recombinant protein (5 μg/well) and incubated with 100 μl of serially diluted mice serum or TB-PBL’s serum. Plates were then incubated with mouse anti-human IgG conjugated alkaline phosphatase (AP) (Sigma) and the plate was read using a BioRad microplate reader at an absorbing wave length of 415 nm.

The splenocytes were harvested and pooled 2 weeks after the second vaccination. Human IFN-γ ELISpot assay and the IL-2 ELISpot assay were performed using Human IFN-γ ELISpot Kit (856 051 005PC, Diaclone, Besancon, France) and Human IL-2 ELISpot Kit (856 001 005PC, Diaclone, Besancon, France) according to the manufacturer’s instructions.

### Flow cytometry

Single-cell suspensions of splenocytes were stained with anti-mouse CD3, CD4, CD8, and CD19 antibodies to detect mouse spleen cells. Pooled PBLs were stained with the anti-human antibodies: anti-human CD3 (clone SK7), CD4 (SK3), CD8 (RPA-T8) and CD19 (4G7). All antibodies were obtained from BD Pharmingen (San Diego, CA, USA) cells were analyzed on a FACS Calibur flow cytometer.

### Cytotoxic assays and tumor cell challenge

The non-radioactive cytotoxicity assay (G1708, Promega, USA) was used to quantify the release of a cytosolic enzyme, lactate dehydrogenase (LDH) upon the lysis of the target cell. Prior to the tumor cell challenge, pooled splenocytes (effector cells, E) were incubated with the M1H, M3H, MMH recombinant protein (5 mg/ml) for 2 h at room temperature. The tumor cells (1 × 10^4^ cells/well) were added to a serial-diluted splenocytes suspension for 4 h at 37 °C. After centrifugation, 50 μl aliquots of the cell-free supernatant were assayed for the LDH content. To correct the confounding effect of spontaneous LDH release from effector cells, the LDH levels were measured at each individual effector cell concentration and used in the real experimental setups (effector spontaneous). The spontaneous release of LDH from target cells (target spontaneous) was also measured. The maximum target cell LDH release level (target maximum) was measured after lysing cells with 0.4 % Triton X-100. This level was defined as a 100 % LDH release. The percentage of specific LDH release was determined with the following formula:

$${\text{\% cytotoxicity}} = \frac{{{\text{Experimental}} - {\text{Effector spontaneous}} - {\text{Target spontaneous}}}}{{{\text{Target maximum}} - {\text{Target spontaneous}}}}$$

In the tumor growth and survival experiment, 3 weeks after the last vaccination, mice were challenged subcutaneously in the groin with 1 × 10^6^ HepG2, A549 and HHCC tumor cells.The tumor growth rate was monitored twice a week by caliper measurement. Tumor volumes (TV) were calculated as follows: TV = length × width^2^ × π/6. A death event was defined as having to sacrifice an animal due to excessive tumor burden, tumor abscess, or unresponsiveness to stimuli.

### Statistical analysis

Survival curves for tumor-free survival were plotted using Kaplan–Meier method and difference between groups was analyzed using the log-rank test. Cytokine responses were presented as mean ± SD. One way analysis of variance (ANOVA) with a Newman-Keuls post hoc test was used to compare the difference among three groups and the difference between specific two groups. Tumor sizes were analyzed using the Mann–Whitney-U test. Statistical analyses were performed using GraphPad Prism software. For all analyses, two-tail p values below 0.05 were defined as significantly different.

## Results

### Recombinant proteins expression, identification and detection in tumor cell lines

**Fig. 1:**
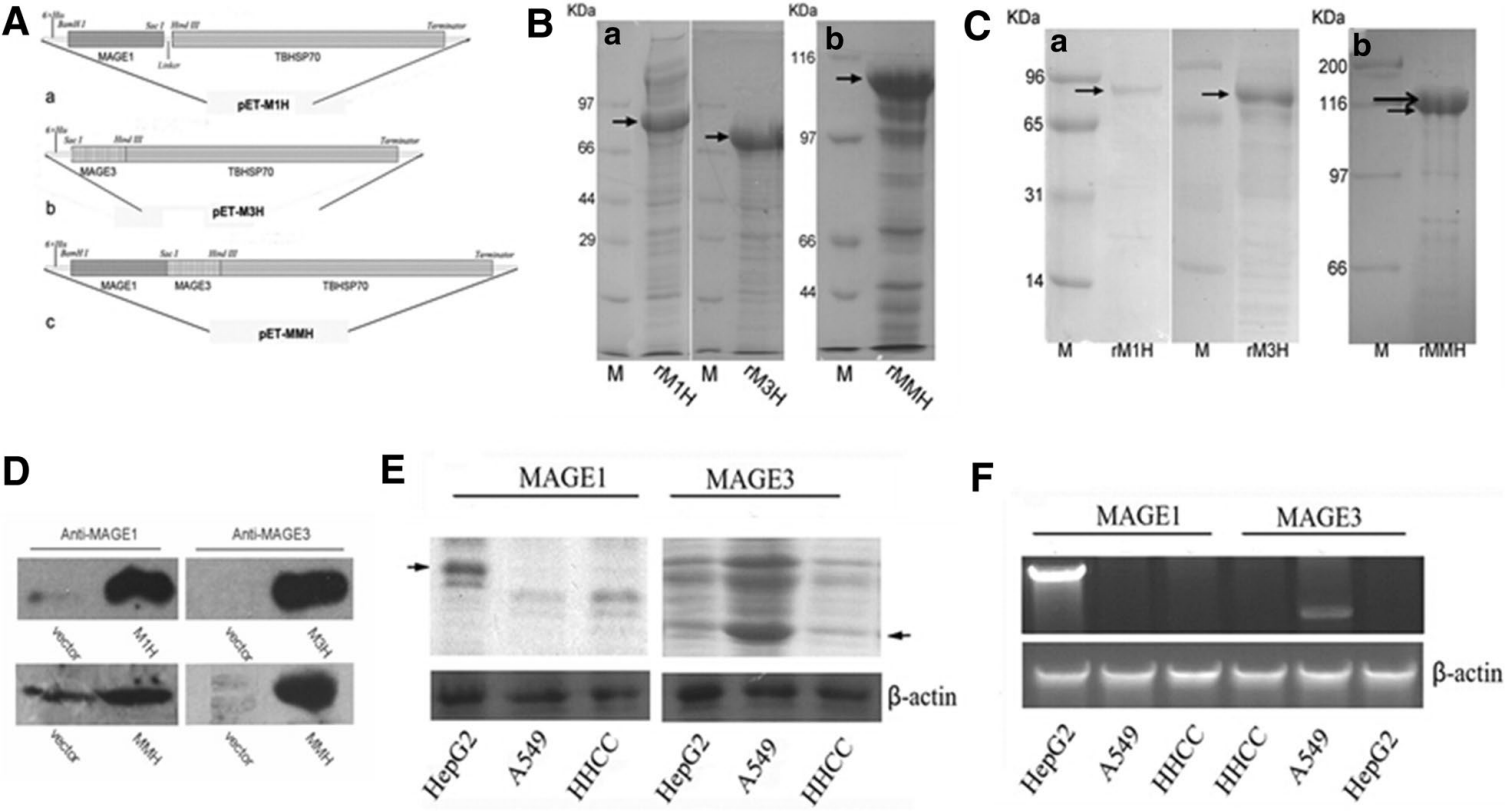
Vaccine information and MAGEs expression in tumor cell lines. **A** The expression vector for the recombinant vaccine protein including MAEG1-TBHSP70 expression vector (*abbr.* pET-M1H), MAGE3-TBHSP70 vector (*abbr.* pET-M3H) and MAGE1-MAEG3-TBHSP70 (*abbr.* pET-MMH) expression vector. **B** Protein lysates were run through electrophoresis gel and stained with Coomassie. **C** Purified recombinant proteins were run through electrophoresis gel and transferred into PVDF membrane, and then blotted with MAGE1 and MAGE3, showing similar pattern as coomassie staining. **D** Purified recombinant protein were blot with MAGE1 and MAGE3 separately. **E** Protein level of MAGE1 and MAGE3 in three different tumor cell line including HepG2, A549 and HHCC. **F** mRNA transcription levels of MAGE1 and MAGE3 in HepG2, A549 and HHCC cell lines using nest-RT-PCR

The expression proteins sequence were predicted as model shown in Fig. 1A, while the molecular weight were calculated 91,918 Da of M1H, 8,109,861 Da of M3H and 106,113 Da of MMH by software. Accessed by western blot Gel-Pro Analysis software Image-Pro Premier (MediaCybernetics Inc., MD, USA), the molecular weight of M1H, M3H1 and MMH were 92, 86 and 110 kDa, respectively (Fig. 1B). Using anti-MAGE1 and anti-MAGE3 antibodies as a probe for western blot, the recombinant proteins M1H and M3H both showed epitopes for MAGE1, while rMMH had epitope for both MAGE1 and MAGE3 (Fig. 1B, C). The recombinant proteins were further analyzed by mass spectrometry, the sequences and molecular mass were both consistent with the prediction (data not shown).

We further study whether commonly used tumor cell lines including HepG2, A549 and HHCC expressed MAGEs family members including MAGE1 and MAGE3. HepG2 cells express a 45 kDa protein when blotted with anti-MAGE1 antibody (Fig. 1E), but not anti-MAGE3 antibody. In contrast, A549 tumor cell lines express MAGE3 protein with a 48 kDa molecular weight, but not MAGE1 (Fig. 1E). HHCC cell line expresses neither MAGE1 nor MAGE3 (Fig. 1D). The protein expression data is consistence with mRNA transcription levels of MAGE1 and MAGE3 in the three different tumor cell lines (Fig. 1F). Briefly, nest-RT-PCR showed MAGE1 mRNA in HepG2 cell line, MAGE3 mRNA in A549 cell line and neither of these mRNAs in HHCC cell line (Fig. 1F). Both protein and mRNA level of MAGE1 and MAGE3 in these three tumor cell lines were consistent with the report from others [13, 16]. Thus, we concluded that HHCC cell neither expressed MAGE-1 nor MAGE-3, while HepG2 cells highly expressed MAGE1 and A549 cells expressed MAGE3.

### Vaccines boosted splenocytes-specific CTL proliferation and recognition but lacks humoral response

**Fig. 2:**
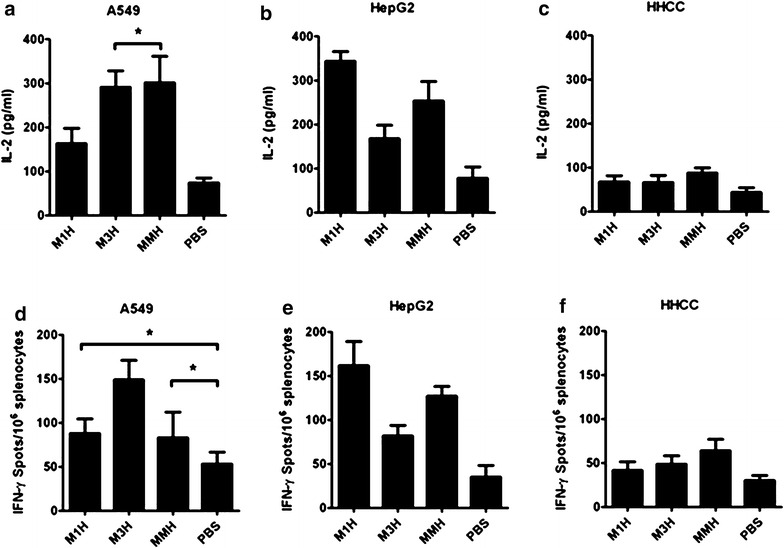
Cellular immunity from splenocyte after stimulation. **a**–**c** IL-2 levels from mice vaccinated with rM1H, rM3H, MMH and PBS and challenged with A549 (**a**), HepG2 (**b**) and HHCC (**c**) cells. **d**–**f** IFN-γ levels from mice vaccinated with rM1H, rM3H, MMH and PBS and challenged with A549 (**d**), HepG2 (**e**) and HHCC (**f**) cells

Stimulated splenocytes from vaccine-immunized mice with tumor cells induced an increased secretion of IFN-γ and IL-2 (Fig. 2). Upon A549 cell stimulation, the splenocytes isolated from rM3H and rMMH vaccinated mice secreted higher level IL-2 than PBS control group (p = 0.0003) (Fig. 2a), while the increased IFN-γ secretion only existed in splenocytes from mice received rM3H vaccine (p < 0.05) (Fig. 2d). The same phenomena were also observed in splenocytes from mice received rM3H vaccine after HepG2 cell stimulation (Fig. 2b, e). After co-incubation splenocytes with HepG2 lysates, the splenocytes from mice receiving rM1H vaccine showed a significantly increased secretion of cytotoxic cytokine including IL-2 and IFN-γ (Fig. 2b, e). In contrast, the splenocytes from mice receiving rMMH vaccine did not show an increased cellular immunity to both HepG2 and A549 stimulation in vitro (Fig. 2c, f). The rMMH induced secretion of cytokines after HepG2 and A549 cells stimulation implied a successful initiation of CTL recognition of the MAGE portions upon the challenge with different tumor cell lines.

**Fig. 3:**
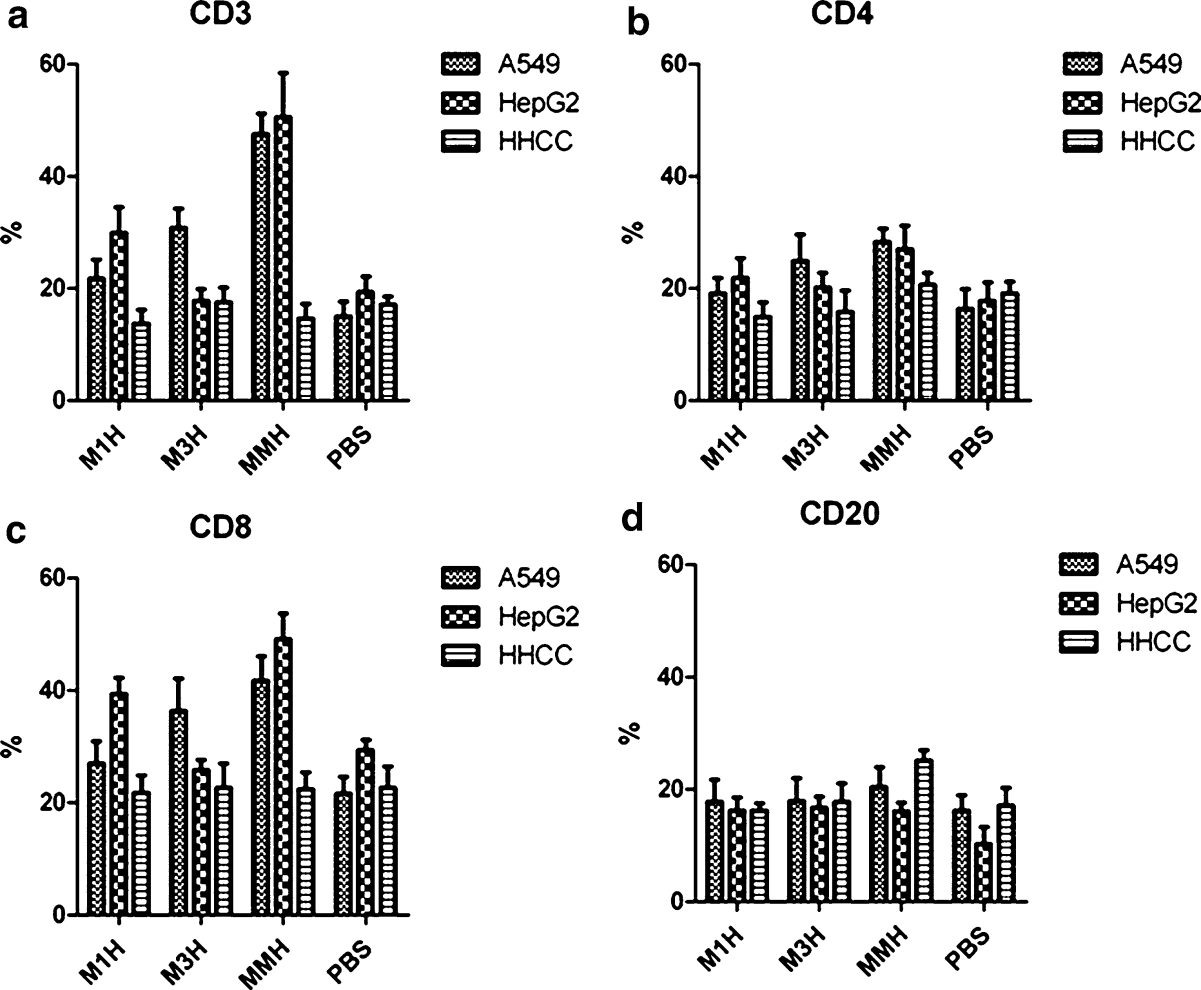
T cell sub-population profile of mice before and after recombinant protein vaccination. **a** CD3+ T cells, **b** CD4+ T cells, **c** CD8+ T cells and **d** CD20+ cells

We further used flow cytometry to investigate the T cell activation and proliferation in these vaccine-immunized mice when challenged with different tumor cell lines. Mice had 18 % CD3+ cells, 17 % CD4+ cells and 20 % CD8+ cells before vaccination (PBS control group in Fig. 3a–d). After being vaccinated by rM1H, CD3+ cells percentage increased to 21 % (Fig. 3a), while CD4+ cells increased to 19 % (Fig. 3b), CD8+ cells increased to 25 % (Fig. 3c) in A549 cell challenged cells. Similarly, mice received rM3H vaccination had a decreased CD3+ fraction (36 %) (Fig. 3a), but an increased CD4+ fraction (24 %) (Fig. 3b) and CD8+ fraction (37 %) (Fig. 3c). Similar phenomena of T cell sub population profile change was also observed in mice received rMMH vaccine (Fig. 3a–c). We did not observe any difference in CD20 cells profile between either of the groups in the current study (Fig. 3d). Thus, our data provided another evidence of increased T cell activation and increased proliferation in mice vaccinated with the recombinant protein vaccines upon tumor cell challenge.

### Effective and specific cellular cytotoxic-response to tumor cells in vitro

**Fig. 4:**
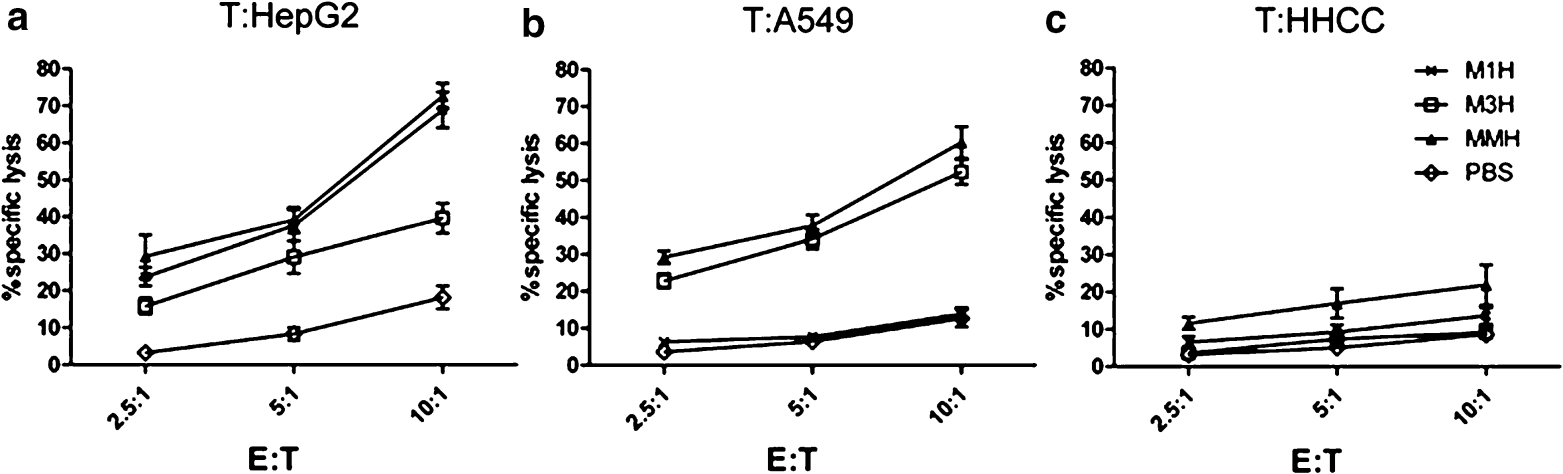
Tumor cell lysis effect induced by CTLs in mice with or without vaccination. **a** After HepG2 tumor cells treatment, **b** after A549 tumor cells treatment and **c** after HHCC tumor cells treatment. *E* effector cells, *T* target cells

To further determine whether recombinant proteins may elicit specific cytotoxicity activity in Hu-PBL-SCID mice ex vivo, splenocytes isolated from vaccine-immunized mice were re-stimulated in vitro with recombinant vaccines or PBS for 2 h at room temperature. The ratio of specific cytolysis to the target cells (E:T ratio) was determined by a nonradioactive cytotoxicity assay. We found that the splenocytes boosted the vaccines efficiency and strengthened the cytotoxic-specific cell lysis effect to both HepG2 and A549 tumor cells chllenge in vitro (Fig. 4a, b). The rM1H and rMMH both induced the attack of immune system towards the tumor cells including HepG2 and A549 tumor cells (p < 0.001) (Fig. 4a, b). In contrast, the specific elimination of HHCC tumor cells was not obvious in mice receive either vaccine or placebo (p < 0.001) (Fig. 4c). These data suggested a role of specific tumor cell lysis effect of recombinant vaccine-immunized mice from the splenocytes, including cell lysis effect of immune system in rM1H-immunized mice toward HepG2 tumor cells, rM3H-immunized mice towards A549 cells and rMMH-immunized mice towards both HepG2 and 549 tumor cells. We did not observe a significant change of HHCC tumor cell lysis in mice received rMMH vaccination (Fig. 4c). This is consistent with our finding that HHCC did not express MAGE1 and MAGE3 which consequently could not induce cellular cytotoxic-response and anti-tumor lysis effect through MAGE family proteins upon HHCC challenge.

### Vaccination protected animals from progressive tumors growth and higher survival rate after the in vivo tumor cell challenge

**Fig. 5:**
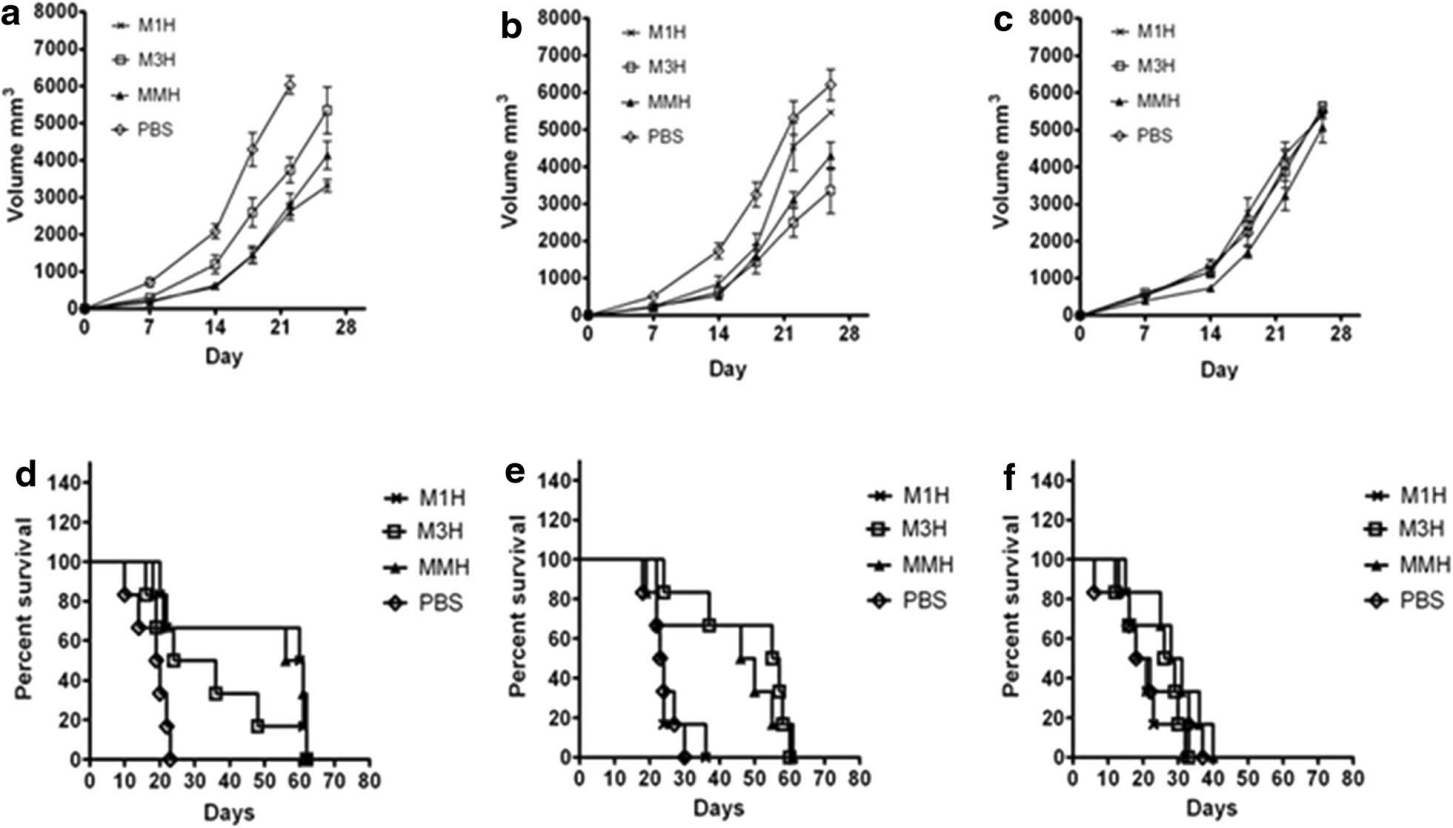
Tumor growth curve reflected by tumor volume and Kaplan–Meier survival curve. **a**–**c** Tumor volume of rM1H, rM3H, MMH and PBS mice challenged with HepG2 (**a**), A549 (**b**) and HHCC (**c**) cells. **d**–**f** Kaplan–Meier survival curve of rM1H, rM3H, MMH and PBS mice challenged with HepG2 (**d**), A549 (**e**) and HHCC (**f**) cell lysate

We carefully measure the tumor volume and follow-up the survival rate of mice in all groups for a period of 60 days after the implantation of tumor cells into these mice. In PBS group, mice died due to extensive tumor load and consequent infection on day 14 before the experimental endpoint (Fig. 5a, b). The tumor growth rate and survival rate were summarized in Table 1 and Fig. 5. Mice received recombinant proteins vaccine rM3H and rMMH had a significantly decelerated tumor growth rate and better survival rate (Fig. 5a, b, d and e and Table 1). In details, the rM1H and the rM3H vaccinated mice had a significantly less degree of HepG2 or A549 cell growing compared with those without vaccination (both p < 0.05), whereas the rMMH-immunized mice was defective in both HepG2 and A549 tumor growth (both p < 0.05) (Fig. 5a, b). This suggested that the rMMH vaccination induced the bi-antigen-specific immune response in vivo and had a protective effect against MAGE-1 or MAGE-3 positive tumors. The median survival days of rMMH-immunized mice treated with either HepG2 or A549 tumor cells was the longest among all groups (Fig. 5d, e and Table 1), strongly suggesting a survival-benefit effect of either MAGE1 or MAGE3 vaccination in mouse. No difference was observed in either tumor cell growth rate or the survival rate in mice challenged with HHCC and PBS (Fig. 5c, f).

**Table 1:** Median survival of experiment mice (days)

	HepG2 (n = 6)	A549 (n = 6)	HHCC (n = 6)
rM1H	60.5 ± 18.8	23 ± 4.9	19.5 ± 5.7
rM3H	30 ± 16.5	56 ± 13	29.5 ± 7.6
rMMH	58.5 ± 19.3*^$^	53 ± 15*^	29.5 ± 8.6*
PBS	19.5 ± 4.6	20.5 ± 3.7	25.5 ± 4.4
UnHuM	13 ± 3.3	12.5 ± 4.1	9.5

In tumor challenge test, rMMH vaccinated mice presented the obvious survival time with HepG2 and A549 cells transplanting. Even in HHCC group the median survival of rMMH vaccinated mice was superior to others

* p < 0.05 vs. PBS group and UnHuM group

^ p < 0.05 vs. rM1H

^$^ p < 0.05 vs. rM3H group

## Discussion

It is well known that the MAGE family is not only a tumor specific antigen but is associated with tumor invasion, metastasis, and patient survival [17, 18]. MAGE-based cancer vaccines are proposed to exert their anti-tumor cell effect through CTL responses as well as the inhibition of tumorgenesis and metastasis. In this study, we studied whether recombinant MAGE1-MAGE3-TBHSP70 protein vaccine targeting two members of MAGE family proteins at the same time can induce cellular cytotoxic-response toward tumor cells and whether such strategy could be an effective on in decelerate the tumor growth rate and prolong the survival rate in vivo in a PBL-Hu-SCID model.

We found that the truncated forms of MAGE1 and MAGE3 used in this study elicited enough anti-tumor reaction and improved the potency of the therapeutic effects. Comparing with rM1H and rM3H vaccine which target single MAGE family member, the notable feature of rMMH vaccine is that it includes the CTL epitopes of both MAGEA family members, MAGE1 and MAGE3, which could significantly increase immune-surveillance and inhibit tumorigenesis.

We have noted that the molecular weight of the recombinant proteins defined in the present study is significantly different from the mass weight predicted on the basis of the sequence. A possible explanation is that recombinant proteins migrate in an anomalous fashion in polyacrylamide gel. The deduced MAGE amino acid composition reveals a high content in acidic residues, which may reduce Sodium dodecyl sulfate binding and lower the polypeptide mobility during electrophoresis. Posttranslational modifications, e.g., phosphorylation or glycosylation of the protein could also count for the different in the molecular weight.

We observed a difference of spleen immune cell profile between normal human spleen and SCID mice or donor’s PBL. One explanation could be that this is resulted from of the irradiation and bone marrow cell depletion procedure and also could be due to the feature of congenital immuno-deficiency in our mouse models. We did not pursue the further investigation of humoral immunity change since we would like to focus on cellular response in the current study. Besides, we found that the response of immune cells, particularly CD3 cells, by different treatment groups (M1H, M3H and MMH) had a different magnitude. It could be interesting to further demonstrate these change in our future study to unravel the role of these vaccines on immune cells.

HHCC cell line was a commonly used liver cancer line. The MAGE expression of HHCC had never be tested. Through screen, we found that most of the members in MAGE family were not expressed on HHCC. However, HHCC had been regarded as a tumor cell line with aggressive growth rate due to its low differentiate degree and abolished contact inhibition effect. Thus, HHCC should be an ideal experimental control in this study. For a cell line likes HHCC which did not express MAGE proteins, such tumor cells would not be recognized by immune-cells after vaccination, and could be a safe approach in the future clinical practice.

Our results showed that the engrafted immune system could be activated by rMMH vaccine to produce sufficient and specific anti-tumor reaction towards hepatocarcinoma and pulmonary carcinomas both in vivo and in vitro. By using HHCC cell line, the immune response in vitro and in vivo helped us distinguishing the efficiency of the three recombinant vaccines in this study. We found that rMMH vaccine had the most extensive immune reaction after being vaccinated to mice as it targeted both MAGE1 and MAGE3. Therefore, recombined MAGE1-MAGE3-TBHSP70 should be a potential candidate for anti-tumor immunotherapy in the future although further study needs to be carried out. Furthermore, this improved methodology for characterizing antigen profiles in cancer may also be helpful for the future customized anti-tumor immunotherapy.

## Abbreviations

MAGE: melanoma antigen-encoding gene
TBHSP70: tuberculosis heat shock protein 70
rM1H: recombinant MAGE1-TBHSP70
rM3H: recombinant MAGE3-TBHSP70
rMMH: recombinant MAGE1-MAGE3-TBHSP70
SCID: severe combined immunodeficient mouse
Hu-PBL SCID: humanized peripheral blood lymphocytes SCID
SDS-PAGE: sodium dodecyl sulfate polyacrylamide gel electropheresis
MW: molecular weight
