# Erratum to: In vivo enhancement of the MAGE-specific cellular immune response by a recombinant MAGE1-MAGE3-TBHSP70 tumor vaccine

**DOI:** 10.1186/s12935-016-0375-5

**Published:** 2017-01-06

**Authors:** Wang Junwei, Zhang Xiumin, Ye Jing, Yang Shoujing, Li Zengshan

**Affiliations:** 1 The State Key Laborotary of Cancer Biology, Xijing Hospital of the Fourth Military Medical University, Xi’an, 710032 Shanxi China; 2 The Pathology Department, Fourth Military Medical University, ChangLe West Road 17, Xi’an, 710032 Shanxi China

## Erratum to: Cancer Cell Int (2016) 16:45 DOI 10.1186/s12935-016-0317-2

Following publication of the original article [1] it was brought to our attention that the second author name had been misspelt. Please note that the second author name should be “Zhang Xiumin” and not “Zhan Xiumin” as was originally presented.
